# α-linolenic acid inhibits human renal cell carcinoma cell proliferation through PPAR-γ activation and COX-2 inhibition

**DOI:** 10.3892/ol.2013.1336

**Published:** 2013-05-08

**Authors:** LIJUN YANG, JIANLIN YUAN, LIWEN LIU, CHANGHONG SHI, LONGXIN WANG, FENG TIAN, FEI LIU, HE WANG, CHEN SHAO, QIANG ZHANG, ZHINAN CHEN, WEIJUN QIN, WEIHONG WEN

**Affiliations:** 1 Departments of Urology, Fourth Military Medical University, Xi’an, Shaanxi, P.R. China;; 2 Ultrasound Diagnosis, Xijing Hospital, Fourth Military Medical University, Xi’an, Shaanxi, P.R. China;; 3 Laboratory Animals Center, Fourth Military Medical University, Xi’an, Shaanxi, P.R. China;; 4 Department of Urology, Northwestern University’s Feinberg School of Medicine, Chicago, IL, USA;; 5 Department of Immunology, State Key Laboratory of Cancer Biology, Fourth Military Medical University, Xi’an, Shaanxi, P.R. China

**Keywords:** renal cell carcinoma, α-linolenic acid, peroxisome proliferator-activated receptor-γ, cyclooxygenase-2, cell proliferation

## Abstract

ω-3 fatty acids have potential anticancer effects, and consuming food rich in ω-3 fatty acids reduces the human renal cell carcinoma (RCC) risk. However, the direct effect of ω-3 fatty acids on RCC *in vitro* is unknown. In the present study, the effects of α-linolenic acid (ALA), an ω-3 fatty acid, were observed on cell proliferation in the RCC cell line OS-RC-2. The activity and gene expression levels of peroxisome proliferator-activated receptor-γ (PPAR-γ) and cyclooxygenase-2 (COX-2) in the OS-RC-2 cells were measured by ELISA and real-time RT-PCR, respectively, following ALA treatment. ALA (20–80 *μ*M) dose-dependently suppressed the proliferation of the OS-RC-2 cells. PPAR-γ activity and gene expression were significantly increased by ALA at 20 and 40 *μ*M. COX-2 activity and gene expression levels were significantly decreased by ALA from 20 *μ*M. Use of purely the PPAR-γ agonist, rosiglitazone, decreased the proliferation of the OS-RC-2 cells, while ALA induced further suppression of cell proliferation in the presence of rosiglitazone. The COX-2 inhibitor N-(3-Pyridyl)indomethacinamide induced further suppression of cell proliferation in the presence of rosiglitazone. N-(3-Pyridyl)indomethacinamide also suppressed the proliferation of the OS-RC-2 cells. In the presence of N-(3-Pyridyl)indomethacinamide, ALA and rosiglitazone further inhibited OS-RC-2 cell proliferation. In conclusion, ALA inhibits the cell proliferation of the OS-RC-2 human RCC cell line. PPAR-γ activation and COX-2 inhibition serve as two signaling pathways for the inhibitory effects of ALA on RCC cell proliferation.

## Introduction

Renal cell carcinoma (RCC) is the most common type of kidney cancer in adults and the most lethal of all genitourinary tumors. Nephrectomy remains the main method of curative treatment for RCC without distant metastasis (1). The other two main types of anticancer treatment in wide clinical use are radiation and chemotherapy. However, the majority of RCCs are resistant to radiation therapy and chemotherapy (2). Current research is focusing on the development of new methods for RCC therapy.

ω-3 fatty acids have potential anticancer effects. The dietary intake of ω-3 fatty acids is associated with a reduced risk of certain types of cancer in human populations and animal models (3). In a large population-based cohort study with data on long-term diet, it was noted that consumption of ocean fish rich in ω-3 fatty acids reduced the RCC risk by ∼74% (4,5). *In vitro* studies have shown that ω-3 fatty acids inhibit cell proliferation and induce apoptosis in certain types of cancer cells (6,7). However, the direct effects of ω-3 fatty acids on RCC *in vitro* have not been reported.

The anticancer effects of ω-3 fatty acids may be mediated by multiple pathways, including peroxisome proliferator-activated receptor-γ (PPAR-γ) activation. PPAR-γ is a nuclear receptor that regulates lipid homeostasis and is implicated in the pathology of numerous diseases, including obesity and diabetes (8,9). Additionally, studies have demonstrated that PPAR-γ has a significant role in the inhibition of tumor growth, progression and metastasis (10). PPAR-γ is expressed in a number of types of cancer including colon, breast, lung and prostate cancer. PPAR-γ activation inhibits the proliferation of numerous types of cancer cells *in vitro*. It has been demonstrated that fatty acids are natural ligands of PPAR-γ (11,12). Fatty acids may activate PPAR-γ, then inhibit the growth of cancer cells. However, the role of PPAR-γ in RCC growth has not been clarified.

Cyclooxygenase-2 (COX-2) has also been suggested to be involved in the development of cancers. COX-2 is an inducible enzyme involved in inflammatory processes. Increasing evidence indicates that COX-2 inhibition has an important role in the prevention of cancer and in the delay of progression in established cancer. For example, ω-3 fatty acids have been shown to downregulate COX-2 expression and inhibit hepatocellular carcinoma cell growth (13). It has been observed that COX-2 is highly expressed in RCC tissues and that it shows a correlation with pathological features and prognosis in patients with RCC (14–16). The role of COX-2 in the development of RCC is not fully understood.

α-linolenic acid (ALA) is an ω-3 fatty acid that certain diets are rich in and that may be transformed to other ω-3 fatty acids, including eicosapentaenoic acid (EPA) and docosahexaenoic acid (DHA), in the cells. In the present study, the effects of ALA on the proliferation of human RCC cell line OS-RC-2 cells were observed *in vitro,* and the involvement of PPAR-γ and COX-2 was demonstrated in the effects of ALA.

## Materials and methods

### Cell culture

The human RCC cell line, OS-RC-2, was obtained from ATCC (Rockville, MD, USA). The OS-RC-2 cells were cultured in RPMI-1640 medium (Sigma, St. Louis, MO, USA) supplemented with 10% heat-inactivated fetal bovine serum (Sigma). The cells were incubated at 37°C in a 5% CO_2_ humidified incubator. This study was approved by the ethics committee of Fourth Military Medical University, Xi’an, China.

### Cell proliferation assay

The cells were seeded into 96-well plates at a density of 10^4^ cells per well. ALA was added into the medium the next day after seeding. Following 48 h of treatment, BrdU was added with ALA into the medium and incubated for one day. The cells were fixed and denatured by incubation with 100 *μ*l FixDenat for 30 min at room temperature. Following the aspiration of FixDenat, the cells were washed with 1 ml blocking buffer [1% bovine serum albumin (BSA) in phosphate-buffered saline (PBS)], then incubated for 90 min with 100 *μ*l anti-BrdU peroxidase-conjugated antibody. The incubation with anti-BrdU peroxidase-conjugated antibody was terminated by three washes with blocking buffer, and 100 *μ*l 3,3′,5,5′-tetramethylbenzidine (TMB) substrates were subsequently added into the wells. Following 5–10 min of incubation with TMB, 25 *μ*l 1 M H_2_SO_4_ was added to stop the reaction. The absorbance was measured by an enzyme-linked immunosorbent assay (ELISA) reader at a wavelength of 450 nm (Bio-Rad Laboratories, Hercules, CA, USA). The BrdU, antibodies, FixDenat and TMB were provided in the BrdU ELISA kit (Roche, Penzberg, Germany).

### PPAR-γ activity assay

The OS-RC-2 cells were seeded into 100-mm dishes and treated with ALA at various doses for 48 h. Subsequent to the treatment, the cells were collected, then centrifuged at 300 × g for 5 min at 4°C. The pellets were washed with ice-cold PBS/phosphatase inhibitor solution, then treated with hypotonic buffer for 15 min. NP-40 was added and the samples were centrifuged for 30 sec at 4°C. The pellets were resuspended in the extraction buffer for 30 min on ice and centrifuged at 14,000 × g for 10 min at 4°C. The supernatants containing the nuclear extracts were collected. The PPAR-γ activity levels of the OS-RC-2 cells were then measured using a PPAR-γ Transcription Factor kit (Cayman, Ann Arbor, MI, USA) according to the manufacturer’s instructions. Briefly, 10 *μ*l nuclear extract from the samples and 90 ml Complete Transcription Factor buffer were added to the designated wells. Following an incubation overnight at 4°C, the wells were washed and the PPAR-γ primary antibodies were added for 1 h at room temperature, followed by washing and incubation with the secondary antibody for a further 1 h at room temperature. The developing solution was added and the reaction was stopped once the blue color had properly developed. The optical density was read with a microplate reader (Bio-Rad Laboratories) at 450 nm.

### COX-2 assay

The OS-RC-2 cells were seeded into 100-mm dishes and treated with ALA at various doses for 48 h. Following the treatment, the cells were harvested and centrifuged at 1,000 × g for 10 min at 4°C. The cell pellets were homogenized in cold Tris-HCl buffer and centrifuged at 10,000 × g for 15 min at 4°C. The supernatant was collected for the COX-2 assay. The protein levels of COX-2 were assayed using a human COX-2 assay kit from IBL (Gunma, Japan), which is a solid-phase sandwich ELISA. Briefly, the test samples and diluted standards were added into the appropriate wells and incubated for 1 h at 37°C. Subsequent to washing, the labeled antibody solution was added and incubated for 30 min at 37°C. The developing solution was then added and the reaction was stopped once the blue color had properly developed. The absorbance of each well was read with the plate reader (Bio-Rad Laboratories) at 450 nm.

### Real-time RT-PCR

The OS-RC-2 cells were seeded into six-well plates. After reaching 80% confluence, the cells were treated with ALA at various doses for 24 h. Subsequent to treatment, the total RNA was extracted from the cells using Trizol reagent according to the manufacturer’s instructions (Gibco BRL, Grand Island, NY, USA). Reverse transcription was performed in a reaction system containing 1 *μ*g RNA, reverse transcriptase, DNA polymerase and random primers. The cDNA obtained was used to run PCR with pairs of primers. The human primers for the real-time PCR were used as previously reported (16,17). The sequences were as follows: COX-2 forward, 5′-ATC ATT CAC CAG GCA AAT TGC-3′ and reverse, 5′-GGC TTC AGC ATA AAG CGT TTG-3′; PPAR-γ forward, 5′-GCC AAG CTG CTC CAG AAA AT-3′ and reverse, 5′-TGA TCA CCT GCA GTA GCT GCA-3′; and β-actin forward, 5′-GCT CCT CCT GAG CGC AAG T-3′ and reverse, 5′-TCG TCA TAC TCC TGC TTG CTG AT-3′. The specificity of the PCR was checked by analyzing the melting curves. The relative mRNA levels were determined by comparing the PCR cycle threshold cDNA of PPAR-γ and COX-2 with that of β-actin.

### Statistical analysis

All data are presented as the mean ± SEM, The data were analyzed by one-way ANOVA. P<0.05 was considered to indicate a statistically significant difference for all statistical analyses.

## Results

### Effects of ALA on human RCC cell line OS-RC-2 cell proliferation

**Figure 1.**
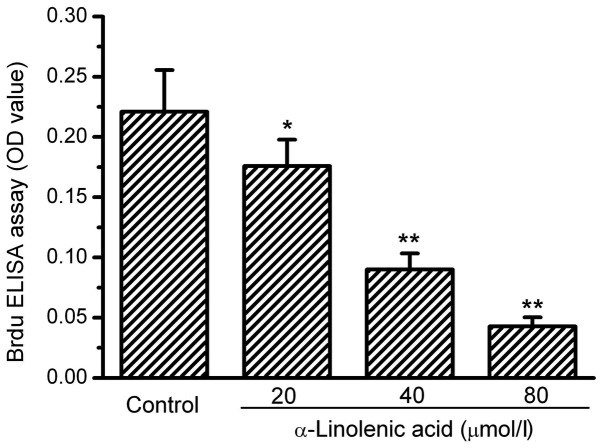
Effects of α-linolenic acid (ALA) on OS-RC-2 cell proliferation as measured by BrdU incorporation. The OS-RC-2 cells were incubated with α-linolenic acid for 48 h and for the following 24 h with the addition of BrdU. Proliferation was reflected by the BrdU incorporation value, which was measured using an ELISA assay with the absorbance read at a wavelength of 450 nm. ALA dose-dependently decreased BrdU incorporation into OS-RC-2 cells. (^*^P<0.05 and ^**^P<0.01 vs. control, n=8). ELISA, enzyme-linked immunosorbent assay.

The human RCC OS-RC-2 cells grew well under the culture conditions. Following 72 h of treatment with ALA at various doses, the cells exhibited a notable suppression of growth. The cell numbers in the groups treated with ALA visibly decreased. To clarify whether the proliferation of the OS-RC-2 cells was affected by ALA, BrdU incorporation into the cells was measured. The results of the BrdU incorporation showed that ALA dose-dependently decreased the incorporation of BrdU into the OS-RC-2 cells, demonstrating an inhibitory action of ALA on OS-RC-2 cell proliferation. The assay values of BrdU incorporation in the 20, 40 and 80 mmol/l ALA treatment groups were 81, 40 and 20% of the control, respectively (Fig. 1).

### Effects of ALA on the activity and expression levels of PPAR-γ and COX-2 in OS-RC-2 cells

**Figure 2.**
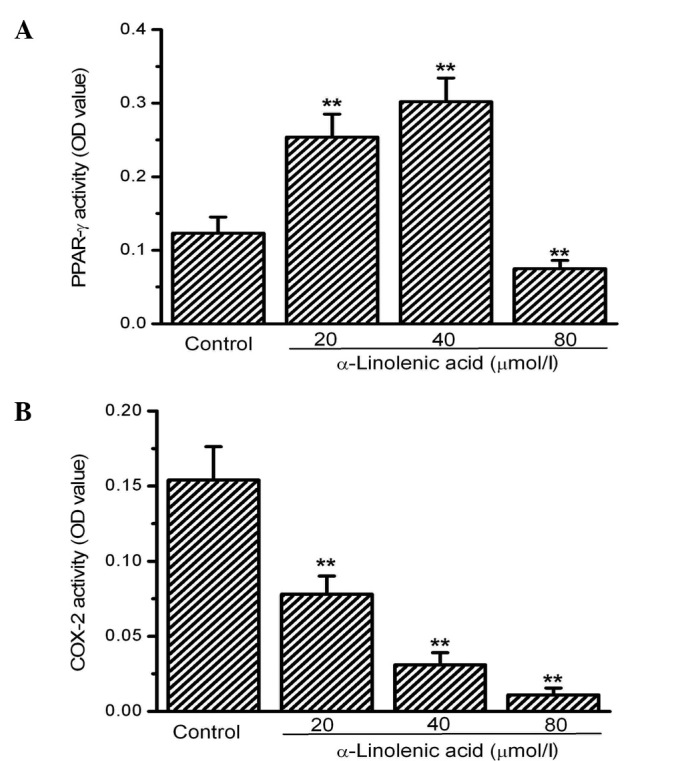
Effects of α-linolenic acid (ALA) on PPAR-γ and COX-2 activity in OS-RC-2 cells. The OS-RC-2 cells were treated with ALA for 48 h and then the PPAR-γ and COX-2 activity were measured via an ELISA assay with the absorbance read at a wavelength of 450 nm. (A) ALA regulated PPAR-γ activity; (B) ALA inhibited COX-2 activity (^*^P<0.05 and ^**^P<0.01 vs. control, n=6). PPAR-γ, peroxisome proliferator-activated receptor-γ; COX-2, cyclooxygenase-2; ELISA, enzyme-linked immunosorbent assay.

Next, the activity of two factors involved in the regulation of tumor growth, PPAR-γ and COX-2, was investigated. PPAR-γ activity was significantly increased by ALA at 20 and 40 mmol/l compared with the control. However, no significant differences were observed between the 20 and 40 mmol/l ALA groups. Notably, 80 mM ALA did not induce an increase in PPAR-γ activity but rather decreased its activity significantly compared with the control (Fig. 2A). In contrast to the activity of PPAR-γ, COX-2 activity was significantly inhibited in the OS-RC-2 cells by ALA. COX-2 activity, measured using an ELISA kit, dose-dependently decreased in the groups treated with ALA compared with the control group (Fig. 2B).

**Figure 3.**
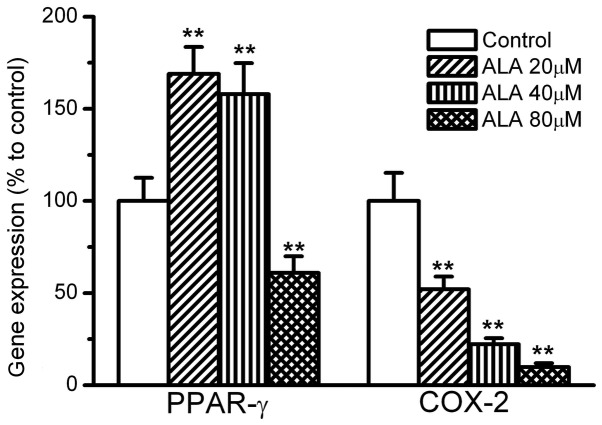
Effects of α-linolenic acid (ALA) on PPAR-γ and COX-2 gene expression. The OS-RC-2 cells were treated with ALA for 24 h, and the PPAR-γ and COX-2 gene expression was measured by real-time RT-PCR. The percentages relative to the control were analyzed. ALA dose-dependently regulated the expression of PPAR-γ and COX-2. (*P<0.05 and ^**^P<0.01 vs. control, n=5). PPAR-γ, peroxisome proliferator-activated receptor-γ; COX-2, cyclooxygenase-2; ELISA, enzyme-linked immunosorbent assay.

The gene expression levels of PPAR-γ and COX-2 were also affected by ALA. ALA at 20 and 40 mmol/l induced >60% greater expression of PPAR-γ compared with the control. However, ALA at 80 mmol/l did not increase the expression of PPAR-γ, and instead decreased it to 60% that of the control. By contrast, COX-2 gene expression was significantly suppressed by ALA. ALA at 20, 40 and 80 mmol/l inhibited the expression of COX-2 to 60, 30 and 10% that of the control, respectively, (Fig. 3).

### Role of PPAR-γ and COX-2 in ALA-inhibited proliferation of OS-RC-2 cells

**Figure 4.**
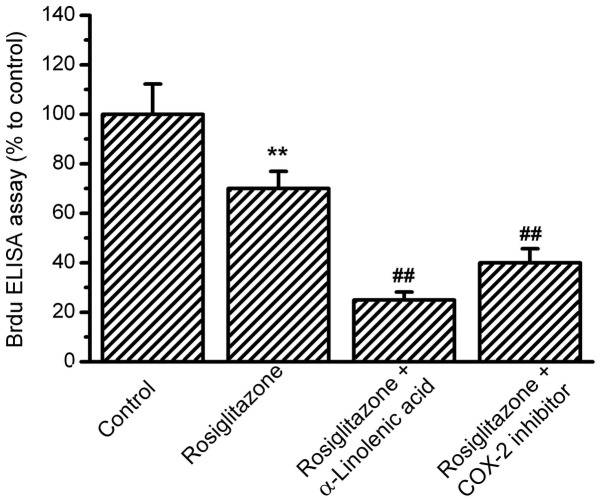
Effects of PPAR-γ activation on OS-RC-2 cell proliferation. OS-RC-2 cells were treated with the agonist of PPAR-γ, rosiglitazone, for 24 h, then treated with α-linolenic acid (ALA) or the COX-2 inhibitor, N-(3-Pyridyl)indomethacinamide. BrdU incorporation was measured by ELISA with absorbance at 450nm. The percentages relative to the control were analyzed. Rosiglitazone inhibited OS-RC-2 proliferation, and in the presence of rosiglitazone, ALA and COX-2 inhibitors further inhibited OS-RC-2 proliferation. (^**^P<0.01 vs. control and ^##^P<0.01 vs. rosiglitazone group, n=8). PPAR-γ, peroxisome proliferator-activated receptor-γ; COX-2, cyclooxygenase-2; ELISA, enzyme-linked immunosorbent assay.

**Figure 5.**
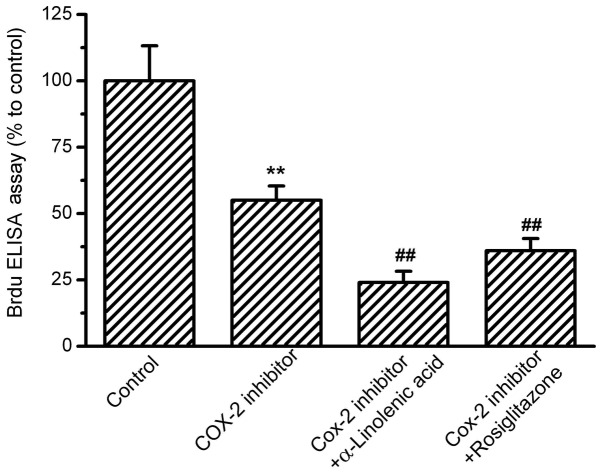
Effects of COX-2 inhibition on OS-RC-2 cell proliferation. The OS-RC-2 cells were treated with COX-2 inhibitor, N-(3-Pyridyl)indomethacinamide, for 24 h, then treated with α-linolenic acid (ALA) or the agonist of PPAR-γ, rosiglitazone. BrdU incorporation was measured by ELISA with absorbance at 450nm. The percentages relative to the control were analyzed. The COX-2 inhibitor decreased OS-RC-2 proliferation and in the presence of the COX-2 inhibitor, ALA and rosiglitazone further inhibited OS-RC-2 proliferation. (^**^P<0.01 vs. control and ^##^P<0.01 vs. rosiglitazone group, n=8). COX-2, cyclooxygenase-2; PPAR-γ, peroxisome proliferator-activated receptor-γ; ELISA, enzyme-linked immunosorbent assay.

To clarify the role of PPAR-γ and COX-2 in the ALA-inhibited proliferation of the OS-RC-2 cells, the effects of a PPAR-γ agonist on BrdU incorporation into OS-RC-2 cells were observed. Rosiglitazone (10^−5^ M), an agonist of PPAR-γ, significantly inhibited BrdU incorporation, supporting the hypothesis that PPAR-γ activation is involved in the inhibition of OS-RC-2 cell proliferation. In combination with PPAR-γ activation by rosiglitazone, ALA at 40 mmol/l inhibited proliferation, indicating that PPAR-γ activation is not the only pathway in ALA-inhibited proliferation. Moreover, at 10^−6^ mol/l, N-(3-Pyridyl)indo-methacinamide, a potent and selective COX-2 inhibitor, suppressed the cell proliferation in combination with PPAR-γ activation by rosiglitazone (Fig. 4). Next, the effects of COX-2 inhibition on OS-RC-2 cell proliferation were investigated. N-(3-Pyridyl)indomethacinamide at 10^−6^ mol/l suppressed the cell proliferation significantly by ∼60% compared with the control. In combination with the COX-2 inhibitor, ALA and the PPAR-γ agonist, rosiglitazone, induced further decreases in proliferation (Fig. 5).

## Discussion

The present study demonstrated that ALA, an ω-3 fatty acid, inhibited the proliferation of human RCC cells *in vitro*, providing direct evidence for the beneficial effect of ω-3 fatty acids on RCC. A previous study showed that ω-3 fatty acids reduce the risk of RCC (5), but no cellular evidence was provided. The present study showed that ALA treatment significantly inhibited RCC cell proliferation by the activation of PPAR-γ and the inhibition of COX-2.

The beneficial effects of ω-3 fatty acids in cancer therapy have been shown in a number of types of cancer. For example, ω-3 fatty acids have been shown to inhibit the growth of breast cancer (18,19) and reduce the risk of lung, prostate and pancreatic cancer (20–23). The present study identified ALA, an ω-3 fatty acid, as an anticancer agent for RCC.

The present study demonstrated that PPAR-γ activation is a signaling pathway that is involved in the action of ALA on OS-RC-2 RCC cells. PPAR-γ is a transcriptional factor that regulates metabolism and numerous other cell functions (24–26). Fatty acids are the natural ligands of PPAR-γ, and ALA is able to activate PPAR-γ (27–29). Therefore, the present study observed the activity of PPAR-γ in OS-RC-2 cells following ALA treatment. As expected, PPAR-γ activity and gene expression were markedly increased by ALA. Furthermore, it was observed that the PPAR-γ agonist inhibited the proliferation of the OS-RC-2 cells. This is supported by studies which reported that the PPAR-γ agonist showed inhibitory effects on cancer growth in certain types of cancers (10,30–32). Taken together, it may be suggested that the activation of PPAR-γ by ALA is one of the signaling pathways for the inhibition of OS-RC-2 cell proliferation.

In combination with the PPAR-γ agonist, ALA further inhibited cell proliferation, indicating that other mechanism besides PPAR-γ were also involved in the action of ALA. The present study clarified that COX-2 inhibition is another mechanism for the inhibition of OS-RC-2 cell proliferation by ALA. COX-2 activity and gene expression were suppressed following ALA treatment. To confirm the role of COX-2 inhibition in the ALA-inhibited proliferation of OS-RC-2 cells, the effects of COX-2 inhibitor on the proliferation of OS-RC-2 cells were investigated. The results showed that the COX-2 inhibitor significantly inhibited OS-RC-2 cell proliferation. Therefore, it was demonstrated that COX-2 inhibition led to decreases in OS-RC-2 cell proliferation. ALA inhibits COX-2 activity, resulting in the inhibition of cell proliferation. Therefore, COX-2 inhibition is considered to be another signaling pathway through which ALA inhibits RCC cell proliferation.

The involvement of COX-2 in cancer cell development has been demonstrated in a number of types of cancer cell (33–35). For example, it has been reported that COX-2 inhibition led to decreases in the proliferation of prostate cancer, MDA-MB-435 cancer and PaCa2 pancreatic cancer cells (36–39). The involvement of COX-2 in RCC has also been suggested. It is reported that COX-2 is highly activated in RCC tissues (16,40,41). The present study demonstrated that COX-2 inhibition suppressed the proliferation of the OS-RC-2 cells, indicating that COX-2 activation is a factor for RCC cell proliferation. Moreover, the present study showed the inhibition of COX-2 by ALA, an ω-3 fatty acid. This demonstrated the direct correlation between ω-3 fatty acid and COX-2.

Therefore, it is suggested that PPAR-γ activation and COX-2 inhibition serve as two signaling pathways for the inhibitory effects of ALA in RCC cell proliferation, and that these two signaling pathways are parallel in the route map. PPAR-γ activation and COX-2 inhibition have synergistic effects on the inhibition of OS-RC-2 cell proliferation. In the present study, following PPAR-γ activation, the COX-2 inhibitor further suppressed the proliferation of the OS-RC-2 cells in addition to PPAR-γ activation, suggesting that COX-2 is not a downstream molecule of PPAR-γ in OS-RC-2 cells. Although it has been suggested that PPAR-γ regulates COX-2 activity in lung cancer (42,43), the present results did not support the activation of COX-2 by PPAR-γ in the OS-RC-2 cells. The discrepancy between the present and previous studies may be due to tissue-specificity. However, in combination with the COX-2 inhibitor, PPAR-γ induced further inhibition of the cell proliferation, indicating that COX-2 is not the regulator of PPAR-γ action. Therefore, we conclude that PPAR-γ and COX-2 are two parallel signaling pathways that mediate the inhibitory effects of ω-3 fatty acid, ALA, on the proliferation of OS-RC-2 cells.

While ALA increased the activity levels of PPAR-γ at concentrations of 20 and 40 *μ*M in the present study, high doses of ALA up to 80 *μ*M inhibited the activity of PPAR-γ. This inhibition may be due to the inhibition, but not the activation, of PPAR-γ gene expression by ALA at 80 *μ*M. The inhibition of gene expression indicates that high doses of ALA have toxic effects on cell viability. The toxic effects of high doses of ALA on the OS-RC-2 cells were not clearly observable through its inhibition of COX-2 expression as the regulated inhibition and toxic expression overlapped. The inhibition of COX-2 expression by ALA ≤80 mM observed in the present study may not be purely due to the regulated inhibitory effects of ALA; the toxic effects of ALA at high doses may be part of the inhibitory action of ALA. It has been demonstrated that high concentrations of certain fatty acids cause cell death via apoptosis or necrosis in numerous types of cells, such as human melanoma cell lines and pancreatic β-cells (44–46). The loss of membrane integrity and the effects on energy metabolism in cells may be two pathways involved in the toxic action of fatty acids. ALA at high doses may affect integrity or inhibit the energy metabolism of OS-RC-2 cells. The exact mechanism of the toxic action of ALA on OS-RC-2 cells remains to be studied.

In conclusion, the present study demonstrated that an ω-3 fatty acid, ALA, inhibited the proliferation of OS-RC-2 cells, a type of human RCC cell line. PPAR-γ activation and COX-2 inhibition are two signaling pathways involved in the action of ALA on OS-RC-2 cells. We suggest that ALA and drugs regulating the activities of PPAR-γ and COX-2 may be potential targets for RCC therapy.
